# Angina bullosa hemorrhagica, an uncommon oral disorder. Report of 4 cases

**DOI:** 10.4317/jced.56840

**Published:** 2020-05-01

**Authors:** Javier Alberdi-Navarro, Abel García-García, Francisco Cardona-Tortajada, María-Luisa Gainza-Cirauqui, José-Manuel Aguirre-Urizar

**Affiliations:** 1 Oral Medicine and Oral and Maxillofacial Pathology Units. Dental Clinic Service, Department of Stomatology II, School of Medicine and Nursing, University of the Basque Country (UPV/EHU), Leioa, Bizkaia, Spain; 2 Oral Medicine, Oral Surgery and Implantology Unit, Faculty of Medicine and Dentistry, University of Santiago de Compostela, Health Research Institute of Santiago de Compostela (IDIS), Santiago de Compostela, A Coruña, Spain; 3 Oral Health Department, Navarre Health Service-Osasunbidea, Pamplona, Spain; 4 Department of Dental Surgery, Faculty of Dental Surgery, University of Malta, Msida, Malta

## Abstract

Angina bullosa hemorrhagica (ABH) is a rare oral disorder characterized by blood-filled bullous lesions in the oral cavity and the oropharynx in the absence of an underlying systemic, haematological or mucocutaneous condition. The presentation of the lesions is acute and located on the lining mucosa, mainly on the soft palate. Often, these lesions are single and rupture easily leaving an ulcerated area. In this study, we present 4 ABH cases in 3 women and 1 man and we discuss the main clinicopathological characteristics. The characteristics of this disorder are important to recognize in order to differentiate the lesions from other oral bullous conditions of the oral cavity such as mucocutaneous disorders or blood coagulation disorders.

** Key words:**Angina bullosa hemorrhagica, angina bullosa haemorrhagica, oral blisters.

## Introduction

Angina bullosa hemorrhagica (ABH) is a rare benign condition characterised by subepithelial blood-filled bullae in the oral or oropharyngeal mucosa, in absence of dermatological, haematological or systemic disorders (1).

The real prevalence and incidence of ABH are unknown although it is considered to be present in 0.05% of patients seen in specialized units of Oral Medicine and Oral Pathology (2,3). Generally, this disorder is seen in adults over 30 years of age, with a peak incidence in the 5th decade of life and without a clear gender predilection (1).

The etiopathogenesis of ABH is yet unknown and therefore considered multifactorial. Trauma on the oral mucosa is considered to precipitate the typical bullous lesions in susceptible individuals (4).

This disorder is characterized clinically by the sudden onset of one or more red-blue blood-filled subepithelial bullae, mainly on the soft palate, lateral borders of the tongue and buccal mucosa (4-6). The time of evolution of the bullae is variable but generally short as they rupture leaving a superficial ulcer that heals within one or two weeks without scarring (1,5,7,8). Recurrent lesions are frequent, appearing on the same or in another location (2,6).

In the rare cases where a biopsy was taken, the histopathological features of ABH were non-specific, presenting as a blood-filled subepithelial bulla with atrophic epithelium and chronic inflammatory infiltrate or as a non-specific superficial ulceration, covered by a fibrinopurulent membrane and chronic inflammatory infiltrate (2,4,9).

Diagnostic criteria for ABH have been suggested recently (Table 1) (6).

**Table 1:**
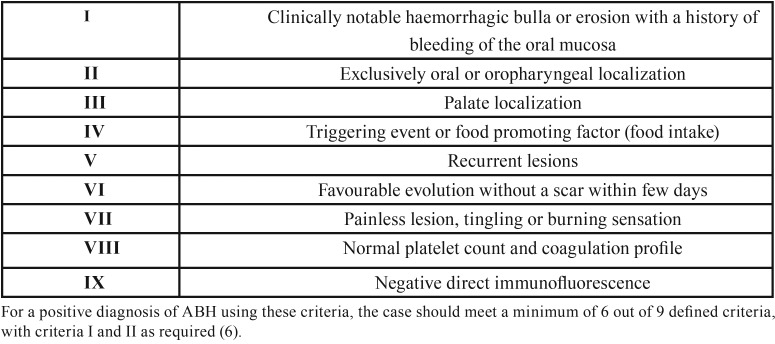
Clinical criteria for the diagnosis of ABH as proposed by Ordoini *et al.*, 2019 (6).

The management for this disorder is symptomatic and focuses on healing of the ulcers and in preventing the onset of new lesions. The overall prognosis of ABH is good, although there are cases where obstruction of the airway has been reported (1,10).

In this paper, we analyze the main clinicopathological characteristics of 4 cases diagnosed as ABH by applying the proposed criteria for this condition and by performing a differential diagnosis with the main blood-filled vesiculo-bullous conditions of the oral cavity.

## Case Report

**Figure 1:**
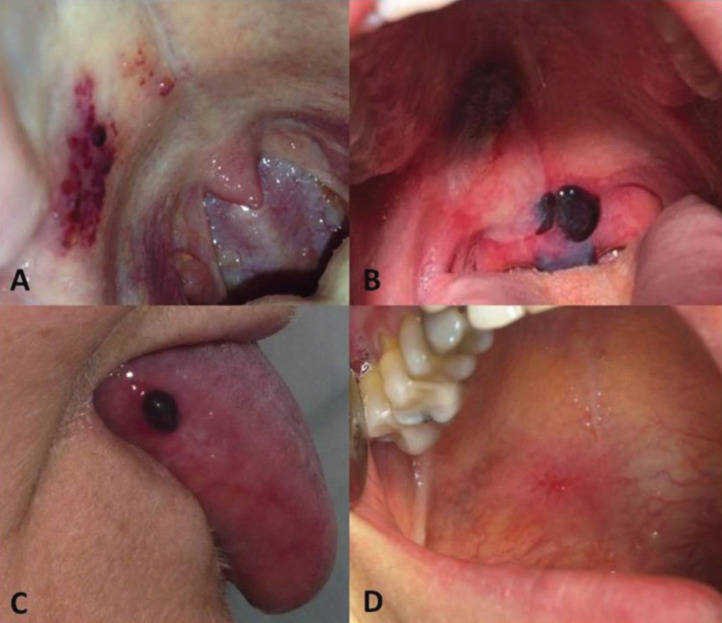
A) Blood-filled vesiculo-bullous lesions on the soft palate. Case 1. B) Bullous lesions on the soft palate and uvula, showing some of the lesions rupture. Case 2. C) Blood-filled bullous lesion on the lateral border of the tongue in case 3. D) Ulcerated area following rupture of the bullous lesion in case 4.

Case 1: A 28-year-old male, with no systemic conditions, toxic habits or known allergies, was seen at the clinic presenting with multiple blood-filled vesiculo-bullous lesions on the right soft palate (Fig. 1A). These lesions were asymptomatic and would appear each time the patient would take a plane, having two previous episodes with similar lesions that healed ad integrum in 2-3 days. There were no other lesions on the skin or other mucosae, no general abnormalities, and the results of the blood tests, including coagulation profile, were normal. Diagnostic criteria of 7/9.

Case 2: A 22-year-old woman presenting to consultation with blood-filled blistering lesions on the soft palate and the uvula of a sudden onset (Fig. 1B). The patient does not refer any other intraoral or extraoral abnormalities. This manifestation has been present twice, with no correlating factors. The bullae last between 1 and 2 days causing discomfort when swallowing and speaking. Once the bullae rupture, the ulcerated lesions healed in a few days without scarring. The results of the blood test, including coagulation profile, were non-significant. Diagnostic criteria of 6/9.

Case 3: A 62-year-old woman presenting with sudden and recurrent blood-filled blisters on the lateral border of the tongue (Fig. 1C). The patient presented with a 10-year history of surgical removal of thyroid cancer currently in treatment with levothyroxine sodium (100 mg/day). The blisters were asymptomatic and ruptured after 2-4 hours, while the succeeding ulcer healed within 7 days without scarring. The patient did not present with any other cutaneous, mucosal or systemic lesions. The results of the blood tests, including coagulation profile, were non-significant. Diagnostic criteria of 8/9.

Case 4: A 49-year-old woman, with no relevant medical history, presenting for the last months with recurrent blood-filled blisters on the soft palate, lateral border of the tongue and buccal mucosa. These lesions ruptured in 3-6 hours leaving an ulcerated surface (Fig. 1D) that caused slight discomfort and healed without scarring in 7 days. Other cutaneous or mucosal lesions were not noticed and the results of the blood test, including coagulation profile, were non-significant. Diagnostic criteria of 7/9.

The main clinical characteristics of the 4 cases are presented in Table 2.

**Table 2:**
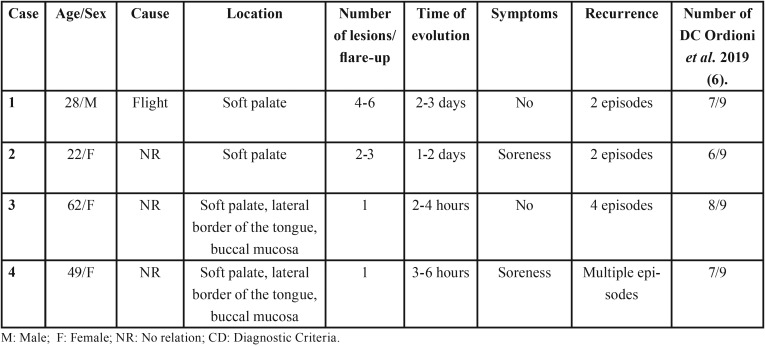
Data of the cases presented in this study.

## Discussion

The ABH is an intriguing benign pathological entity of the oral cavity initially defined by Badham in 1967 (11), although it had been previously described with different names such as hemorrhagic bullous stomatitis, localized oral purpura or stomatopompholyx hemorrhagica (2,9).

The etiology of this benign oral pathology is still unknown and it is considered to occur in susceptible patients following a traumatic event (1,6). Among the described traumatic factors are consuming hard or crunchy food, certain dental treatments or even medical procedures such as an intubation (1,10,12). The onset of ABH has also been linked to certain drug therapies, mainly topical steroids, as well as, although more controversial, with certain systemic conditions such as hypertension and diabetes mellitus (1,6). The use of topical steroids is known to cause epithelial atrophy and alteration of the elastic fibres of the lamina propria, which may promote the formation of subepithelial bullous lesions following trauma (13,14). In our case, none of the patients were being treated with topical or inhaled steroids. We were only able to identify the traumatic cause in one of the cases (Case 1) where the patient referred the association between presenting the bullae and travelling by airplane. This has been previously recorded and we believe it can be associated with atypical movements and/or with changes in environmental pressure causing oral mucosal trauma and leading to the onset of the bullae.

In 2019, Ordoini *et al.*, (6) published a proposal of diagnostic criteria for ABH following a systematic review of previously published cases. According to these authors, the diagnosis of ABH is given if the case fulfills 6 out of 9 of the proposed criteria, with criteria 1 and 2 as required (Table 2). These criteria are clinical in essence and suggest that, although other mucocutaneous and haematological pathologies should be excluded, this should be done only when the clinical presentation of ABH is atypical (6). All of our cases fulfilled the diagnostic criteria as they all met more than 6 criteria. Although we believe that this proposal for diagnostic criteria is the first approach in defining ABH objectively, we consider that further studies are required to analyze their sensitivity and specificity.

An important aspect to consider in ABH is the location of the lesions. As described in other studies (4-6,8), the most common site of appearance of the bullous lesions is the soft palate, and therefore is recognized as a diagnostic criteria for ABH (6). In our case, 3 of the patients presented with lesions in this location. This predominance for the soft palate may be related to a higher risk of trauma during mastication and swallowing, and because of a thin mucosal epithelial lining that may be damaged more than other more protected sites, such as the hard palate and the gingiva.

The differential diagnosis of ABH must be done with vesiculobullous conditions of the oral mucosa, primarily haematological and mucocutaneous conditions of an immunological origin (1,5,6).

Some haematological disorders that may have a clinical presentation similar to ABH in the oral cavity are thrombocytopenia, von Willebrand disease, leukaemia and some vasculitis (9,15). In order to exclude these disorders, it is necessary to perform a thorough medical history and examination, as well as a blood analysis that includes the complete blood count and coagulation profile. In our case, all of our patients presented normal blood test results, including coagulation profile.

The main group of conditions included in the differential diagnosis are mucocutaneous diseases of an immunological origin, including various conditions such as mucous membrane pemphigoid, pemphigus vulgaris, lineal IgA disease, acquired epidermolysis bullosa and bullous amyloidosis (1). These conditions are ruled out by examining other mucosae and skin for other lesions.

We observed a high recurrence rate of ABH lesions in our cases since all patients expressed having previous episodes. This rate is higher than the 62% reported by Ordioni *et al.*, in 2019 (6).

Regarding treatment of this benign condition, we should highlight that currently there is no specific treatment for ABH. Management of the symptoms with topical antiseptics, such as chlorhexidine in concentrations between 0.12% and 0.2% has been described (6). In addition, eliminating irritants or traumatic components that may cause recurrence of the lesions is indicated (2,8).

## Conclusions

We may state that ABH is a rare pathology of the oral mucosa that should be included in the differential diagnosis of blood-filled bullous lesions in the oral cavity. Knowing the characteristics of this oral pathology is key in its diagnosis. The applicability of the proposed diagnostic criteria for ABH should be assessed through further controlled studies.
